# Exploring Biomarkers and Mechanisms of Action of Adaptive Immune Response in Age-Related Macular Degeneration Based on Transcriptomics

**DOI:** 10.3390/biomedicines14051123

**Published:** 2026-05-15

**Authors:** Caijian Xiong, Siqi Zhou, Yingxue Hu, Xinrong Xu

**Affiliations:** Department of Ophthalmology, Affiliated Hospital of Nanjing University of Chinese Medicine, Nanjing 210029, China; 039317119@njucm.edu.cn (C.X.); 20233054@njucm.edu.cn (S.Z.); 202430073@njucm.edu.cn (Y.H.)

**Keywords:** age-related macular degeneration, adaptive immune response, *C3*, *HLA-DOA*, bioinformatics

## Abstract

**Background:** Age-related macular degeneration (AMD) is a common retinal degenerative disease linked to adaptive immune response dysregulation. This study aimed to identify shared immune-related biomarkers and explore their underlying mechanisms. **Methods:** GSE29801 and GSE135092 served as training and validation sets. Adaptive immune response-related genes (AIR-RGs) from MSigDB were intersected with AMD-related differentially expressed genes (DEGs) to identify candidate genes. Machine learning algorithms were applied to screen biomarkers, validated in datasets and a mouse model of choroidal neovascularization by qPCR. A nomogram was constructed and assessed. GSEA and immune infiltration analyses explored mechanisms and immune microenvironment associations. **Results:** A total of 148 DEGs were identified, yielding 15 candidate genes after intersection with AIR-RGs. Machine learning identified *C3* and *HLA-DOA* as potential biomarkers, with their differential expression validated across datasets. A nomogram based on these biomarkers demonstrated good predictive performance for AMD pathology (AUC = 0.795). Biomarkers were associated with some immune-inflammatory pathways. Significant differences in immune cell infiltration were observed between AMD and control groups, with biomarkers positively correlated with differentially infiltrated immune cells, such as natural killer cells. **Conclusions:** The identification of the established biomarker *C3* serves as a proof-of-principle for the analytical approach, rather than a novel discovery, thereby validating the model’s capacity to uncover other critical immune targets. Consequently, *C3* and *HLA-DOA* serve as potential biomarkers for AMD, significantly correlated with disease progression via immune pathways and offering insights for immune-based therapeutic strategies.

## 1. Introduction

Age-related macular degeneration (AMD) is a retinal degenerative disease characterized by chronic inflammation and gradual degeneration of retinal pigment epithelium (RPE) and photoreceptors. In clinical practice, AMD is mainly divided into two subtypes: dry (atrophic) AMD and wet (neovascular) AMD. These subtypes have core pathological features, such as the deposition of lipid-containing drusen and RPE dysfunction, and choroidal neovascularization (CNV) is unique to neovascular AMD [1,2]. AMD is one of the main causes of irreversible blindness in the elderly in developed countries, bringing a huge and growing public health burden, and the global prevalence will rise sharply by 2040 [3]. At present, wet AMD is treated with anti-VEGF, which can delay the decline of vision but cannot completely cure it. Limitations include the need for frequent intravitreal injections, suboptimal response in some patients, and disease recurrence upon termination of treatment. Currently, interventions for dry AMD are largely limited to nutritional supplementation [3,4]. Importantly, existing therapeutic strategies do not primarily target inflammation and immune dysregulation—core pathological mechanisms that are shared hallmarks driving the progression of both dry and wet AMD. This unmet clinical need highlights the urgency of elucidating the molecular mechanisms underlying AMD-associated immune pathology, thereby providing a theoretical basis for novel therapeutic strategies applicable across different disease stages.

Adaptive immune response (AIR) are central to maintaining tissue homeostasis, as they orchestrate precise recognition, activation, and regulation of immune responses against abnormal tissues or pathogens. Dysregulation of AIR contributes significantly to disease progression in AMD. Evidence shows that there is an imbalance of T cell subsets in AMD patients. This imbalance occurs both systemically and locally in the retina. Pro-inflammatory cytokines such as IL-17 produced by Th1/Th17 cells can promote CNV and RPE apoptosis; the decrease in the number or function of regulatory T cells (Treg) can reduce the anti-inflammatory ability [2,5]. In addition, abnormal crosstalk between the complement system and adaptive immunity can aggravate the disease. The *CFH* gene polymorphisms affect the activity of immune cells and also enable the release of inflammatory cytokines, making AMD more likely to occur [6]. The identification of autoantibodies in retinal tissues also indicates a role for B-cell humoral immunity [7,8]. Understanding the adaptive immune mechanism in AMD is the key to elucidating disease etiology and also has the potential for transformation to develop new immune regulation strategies.

To this end, we applied machine learning to integrated transcriptomic data from public AMD repositories, aiming to identify key biomarkers linked to adaptive immune dysregulation. We then explored how these factors function and interact within the retinal immune landscape. Our work provides new molecular insights into AIR in AMD and establishes a theoretical foundation for future immune-targeted interventions.

## 2. Methods

### 2.1. Data Acquisition

The datasets used in this AMD study were retrieved from the Gene Expression Omnibus (GEO, https://www.ncbi.nlm.nih.gov/geo/, accessed on 8 October 2025), with GSE29801 serving as the training set and GSE135092 as the validation set. GSE29801 (GPL4133) contained macular retinal sample data from 32 AMD patients and 28 control individuals [9]. Samples with tissue origins of extrafoveal retina and retinal pigment epithelium were excluded from this dataset. GSE135092 (GPL16791) included macular retinal sample data from 27 AMD patients and 101 control subjects. For this dataset, samples derived from non-macular lesional retina and retinal pigment epithelium were removed [10]. To clarify the disease stages applicable to the identified biomarkers, the sample distribution of AMD subtypes within the public datasets was analyzed. As detailed in Table S1, the 32 AMD samples in the GSE29801 dataset encompass 16 cases of dry/atrophic AMD (comprising 14 ‘dry’ and 2 geographic atrophy [‘GA’]), 8 cases of intermediate macular degeneration (‘MD1’ and ‘MD2’), 4 cases of neovascular or wet AMD (‘CNV’), and 4 cases of mixed advanced AMD (‘GA/CNV’), alongside 28 normal controls. This diverse composition of disease stages provides a fundamental basis for evaluating the roles of biomarkers across different phases of AMD progression.

A total of 765 adaptive immune response-related genes (AIR-RGs) were obtained from the Molecular Signatures Database (MSigDB) (https://www.gsea-msigdb.org/gsea/msigdb, accessed on 9 October 2025) by searching with the term GOBP_ADAPTIVE_IMMUNE_RESPONSE (Table S2).

### 2.2. Analysis of Differentially Expressed Genes (DEGs)

Using the limma package (v 3.56.2) [11], we identified DEGs (*p* < 0.05, |log_2_FC| > 0.5) from the training dataset’s expression matrix. These DEGs were visualized with a volcano plot (ggplot2, v 3.4.1) [12] and a heatmap of the top 10 up-/down-regulated genes (ComplexHeatmap, v 2.16.0) [13]. Candidate genes were finally identified by intersecting the DEG list with AIR-RGs using ggvenn (v 0.1.9) [14].

### 2.3. Protein–Protein Interaction (PPI) Network Construction and Enrichment Analysis

The search tool for the retrieval of interacting genes/proteins (STRING) database (https://cn.string-db.org/) was used to map the interaction relationships among the proteins of the candidate genes by generating a PPI network, applying an interaction score cutoff of >0.7. Visualization was performed using circlize (v 0.4.16) [15].

Using the clusterProfiler package (v 4.15.0.003) [16], the candidate genes were subjected to enrichment analyses against the Gene Ontology (GO) and Kyoto Encyclopedia of Genes and Genomes (KEGG) databases to identify significantly associated biological processes and pathways (adjusted *p* < 0.05).

### 2.4. Machine Learning and Gene Expression Level

Employing the training dataset, candidate genes were further screened via least absolute shrinkage and selection operator (LASSO) (using the glmnet package, v 4.1-8) [17], support vector machine-recursive feature elimination (SVM-RFE), and Boruta analyses to pinpoint key AMD-related genes. The optimal lambda value was determined as the one corresponding to the minimum lambda, and genes selected under this condition were designated as LASSO genes. Candidate genes were screened using the SVM-RFE algorithm from caret (v 6.0-94) [18], with the 5-fold cross-validation conducted. The model achieved its lowest error rate and highest accuracy on a specific set of genes; therefore, these were selected as the SVM-RFE gene set. Under default parameters, the Boruta feature selection algorithm was implemented on candidate genes using Boruta (v 8.0.0) [19]. A p-value threshold of 0.01 was set, and a maximum of 100 iterations was specified. A feature was considered to be of significance when its importance was significantly higher than that of its corresponding shadow feature. Conversely, a feature was labeled as irrelevant if its importance was significantly lower than that of its corresponding shadow feature. Subsequently, the confirmed features were designated as Boruta genes. Genes common to the LASSO, SVM-RFE, and Boruta results were identified as intersection genes using a Venn diagram (ggvenn v 0.1.9). Biomarkers were defined by a stringent dual-dataset criterion: only intersecting genes showing consistent expression trends and significant differences (*p* < 0.05, Wilcoxon rank-sum test) between AMD and control groups in both datasets were selected.

### 2.5. Construction and Evaluation of a Nomogram

Based on the GSE29801, a predictive nomogram integrating all biomarkers was built using the rms package (v 6.8-1) [20], in which each biomarker was assigned a score to calculate a total risk sum predictive of AMD incidence. Model calibration (Hosmer-Lemeshow test, *p* > 0.05 via regplot v 1.1) [21] and clinical utility (Decision Curve Analysis (DCA) via ggDCA v 1.1) [22] were assessed. Predictive accuracy was confirmed by Receiver Operating Characteristic (ROC) analysis (pROC v 1.18.5) [23], yielding an Area Under the Curve (AUC) > 0.7, where higher values indicate stronger performance. The predictive performance of the nomogram was further assessed in the independent validation dataset GSE135092.

### 2.6. Gene Set Enrichment Analysis (GSEA)

Spearman correlation analysis was employed to investigate the pathways associated with the biomarkers. Using all samples from the training dataset, correlation coefficients between each biomarker and the remaining genes were derived respectively via the cor function in the stats package (v 4.3.3) [24]. Genes were ranked in descending order based on their correlation coefficients. Using the “c2.cp.kegg_legacy.v2025.1.Hs.symbols” gene set (MSigDB) as reference, biomarker pathway enrichment was characterized through GSEA executed by clusterProfiler (v 4.15.0.003); significance thresholds were set at adjusted *p* < 0.05 and |NES| > 1.

### 2.7. Predictive Analysis

Functional associations for the identified biomarkers were analyzed via the Gene Multiple Association Network Integration Algorithm (GeneMANIA, http://genemania.org, Homo sapiens) database. Their chromosomal positions were then mapped with RCircos (v1.2.2) [25], and subcellular localization was predicted using scores from the GeneCards database (https://www.genecards.org/).

### 2.8. Analysis of Immune Cell Infiltration

The mutual regulation between AIR and innate immunity affected the amplification of inflammatory signals and the process of tissue damage, which were jointly involved in the pathological development of AMD [26]. The ssGSEA algorithm (GSVA v 1.53.28) [27] was first applied to the training dataset to quantify immune infiltration, obtaining the proportions of 28 immune cell types [28] in AMD and control groups. Subsequently, differential infiltration between groups was analyzed with the Wilcoxon rank-sum test (*p* < 0.05), followed by Spearman correlation analyses. These analyses, conducted using the psych package (v 2.4.6.26), assessed relationships among differentially infiltrated immune cells and between these cells and the biomarkers, applying thresholds of |correlation coefficient (cor)| > 0.3 and *p* < 0.05.

### 2.9. Animal Experiment and Quantitative Polymerase Chain Reaction (qPCR)

All animal experiments were approved by the Institutional Review Board of the Affiliated Hospital of Nanjing University of Chinese Medicine (Approval No. 2023NL-KS183) and conducted in accordance with the National Institutes of Health (NIH) Guidelines for the Use of Laboratory Animals. Ten male C57BL/6 wild-type mice, aged seven months, were purchased from Jiangsu Huachuang Xinnuo Co., Ltd. (License No.: SCXK(Su)2025-0012). Using a random number table, the mice were divided into 2 groups: a normal control group and a model group. Mice designated for the model group were anesthetized via intraperitoneal injection of 1.25% avertin (0.2 mL/10 g), their pupils were dilated, and they then underwent retinal photocoagulation. This was delivered by a krypton yellow laser (568 nm) with parameters set to a 100 μm spot size, 0.1 s duration, and 100 mW power. Effective photocoagulation was confirmed by the appearance of bubbles indicating Bruch’s membrane rupture [29]. Five laser spots were applied around the optic disk in each eye. Two weeks post-modeling, mice were euthanized, and retinal-choroidal tissues were collected. Total RNA was extracted using TRIzol reagent (Vazyme, Nanjing, China) and reverse transcribed into cDNA using HiScript III All-in-one RT SuperMix (Vazyme). Reverse transcription-quantitative polymerase chain reaction (RT-qPCR) was carried out with ChamQ SYBR qPCR Master Mix (Vazyme) in a total volume of 20 μL. The reaction system was as follows: 10 μL of 2× Master Mix, 0.8 μL of primers (0.4 μL each forward and reverse), and 9.2 μL of diluted cDNA. The amplification program was: 95 °C for 30 s, followed by 40 cycles of 95 °C for 10 s and 60 °C for 30 s. Relative expression was analyzed using the 2^−ΔΔCt^ method with β-actin for normalization. The primer sequences used in this study are presented in Table 1. Primer specificity was confirmed by a single peak in the melt curve analysis.

### 2.10. Statistical Analysis

GraphPad Prism (v 9.5) and R software (v 4.2.2) were used for data analysis and bioinformatics analysis. For comparisons involving continuous variables between two groups, the data were first assessed for normality using the Shapiro–Wilk test and for homogeneity of variances using the F test. For data that simultaneously satisfied the assumptions of normal distribution and homogeneity of variances, an independent samples Student’s *t*-test was employed for comparison, with results expressed as mean ± standard deviation. For data that did not meet the assumption of normal distribution, the Mann–Whitney U test was used. Differences between groups were evaluated via the Wilcoxon rank-sum test, which was used for bioinformatics analysis. Differences between groups were evaluated via the Wilcoxon rank-sum test, with statistical significance established at *p* < 0.05. *, *p* < 0.05; **, *p* < 0.01; ***, *p* < 0.001; ****, *p* < 0.0001.

## 3. Results

### 3.1. Screening and Functional Enrichment of Candidate Genes

**Figure 1:**
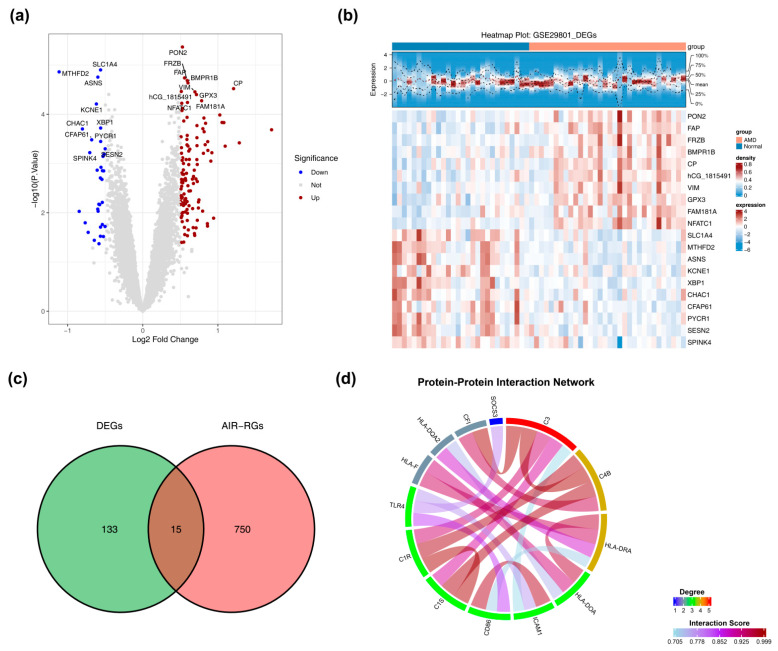
Identification of candidate genes and protein–protein interaction (PPI) network. (**a**) Volcano plot and (**b**) heatmap visualizing the differentially expressed genes (DEGs) between AMD and normal control samples. (**c**) Venn diagram illustrating the intersection of DEGs with adaptive immune response-related genes. (**d**) PPI network demonstrating the interactions among the overlapping candidate genes.

To further screen for genes, DEGs analysis was performed. First, 148 DEGs were identified between the AMD group and the control group (*p* < 0.05, |log_2_FC| > 0.5). Analysis revealed differential expression of these genes in AMD, specifically with 115 upregulated and 33 downregulated (Figure 1a,b and Table S3). Overlapping the 148 DEGs with 765 AIR-related genes identified 15 candidate genes (Figure 1c and Table S4). To further refine these candidates, a PPI network analysis was introduced. Based on network connectivity and a stringent interaction score threshold (>0.7), two genes lacking sufficient interaction evidence were excluded. Consequently, 13 core candidate genes were ultimately filtered and integrated into the network. The PPI network of candidate genes (interaction score > 0.7) revealed that *C3* and *C4B*, among others, served as highly interconnected hubs, demonstrating close interaction relationships with multiple other genes (Figure 1d). Candidate gene enrichment analysis (adjusted *p* < 0.05) identified 321 GO terms, comprising 262 in Biological Processes (e.g., adaptive immune response via somatic recombination, leukocyte/lymphocyte mediated immunity), 33 in Cellular Components (e.g., early endosome, MHC protein complex, blood microparticle), and 26 in Molecular Functions (e.g., peptide binding, peptide antigen binding, antigen binding) (Figure S1a and Table S5). In total, 35 KEGG signaling pathways were significantly enriched. Major representatives were Complement and coagulation cascades, Th17 cell differentiation, and the NF-kappa B signaling pathway, among others (Figure S1b and Table S6). The AIR-related biological processes enriched by these candidate genes were observed to be highly consistent with the pathological characteristics, including immune cell infiltration and abnormal release of inflammatory factors, in the macular retinal tissues of AMD patients. Key links, such as complement system activation and antigen presentation, were regulated by these processes, through which the immune regulatory effect of AIR was precisely anchored in the macular damage process of AMD. This provided a foundation for elucidating the occurrence and development of AMD driven by AIR dysfunction.

### 3.2. Screening of Biomarkers

**Figure 2:**
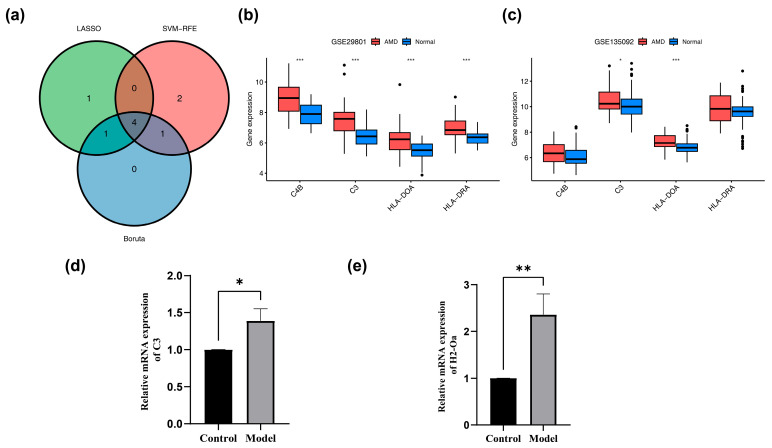
Machine learning-based screening and expression validation of core biomarkers. (**a**) Venn diagram showing the overlapping robust biomarkers identified by LASSO, SVM-RFE, and Boruta algorithms. (**b**,**c**) Expression levels of *C3* and *HLA-DOA* in the training (GSE29801) and validation (GSE135092) datasets, respectively. * *p* < 0.05, *** *p* < 0.001 compared with the control group. (**d**,**e**) RT-qPCR validation of *C3* and *H2-Oa* mRNA expression in the mouse laser-induced CNV model compared to normal controls. Data are presented as mean ± SD. Statistical significance was determined by one-way ANOVA. * *p* < 0.05, ** *p* < 0.01 compared with the control group.

To identify biomarkers closely associated with the pathogenesis of AMD, the 13 core candidate genes finalized in the preceding PPI analysis were subjected to further multidimensional screening to deeply explore their roles in the disease. Machine learning algorithms were first performed on these 13 candidates, and 6 LASSO genes (*C4B*, *C3*, *HLA-DOA*, *HLA-DRA*, *HLA-F*, and *CD86*) were obtained through LASSO analysis [log(lambda min) = −2.7818] (Figure S2a). Through SVM-RFE analysis, 7 SVM-RFE genes (*C3*, *HLA-DRA*, *C4B*, *C1R*, *HLA-DOA*, *C1S*, and *RFTN1*) were selected (Figure S2b). A total of 6 Boruta genes (*C4B*, *C3*, *HLA-DOA*, *C1R*, *HLA-DRA*, and *CD86*) were obtained through Boruta analysis (Figure S2c). After taking the intersection of the three sets, 4 intersection genes (*C3*, *HLA-DOA*, *HLA-DRA*, and *C4B*) were obtained (Figure 2a). *C3* and *HLA-DOA* were significantly up-regulated in AMD patients compared to controls. The result held consistently in both the training and validation sets (*p* < 0.05), as shown by our intersection gene analysis (Figure 2b,c).

To evaluate functional equivalence for the human-specific biomarker *HLA-DOA* in the mouse model, we measured mRNA levels of its ortholog *H2-Oa* [30]. The relative expression level of *C3* mRNA in the retinal tissue of the model group (1.389 ± 0.161) was significantly higher than that in the control group (1.001 ± 0.001) (t = 4.17, *p* = 0.014). Similarly, the relative expression level of *H2-Oa* mRNA (2.360 ± 0.445) was also significantly higher than that in the control group (1.003 ± 0.002) (t = 5.29, *p* = 0.006) (Figure 2d,e). The successful extraction of *C3*—a gene with a well-established association with AMD—served effectively as a positive control. This successful replication of a known pathogenic link provides solid proof-of-principle validation for the reliability of the employed machine learning modeling approach. Consequently, *C3* and *HLA-DOA* were selected as biomarkers for the following analyses.

### 3.3. Predictive Accuracy of Biomarkers for AMD

**Figure 3:**
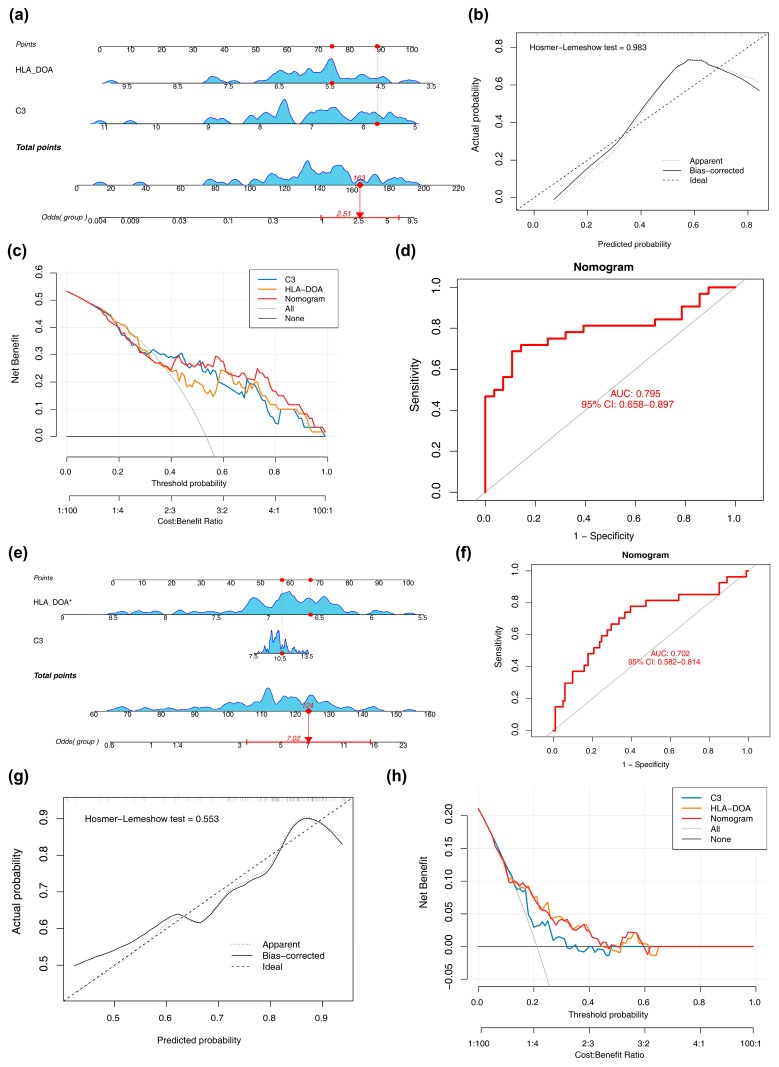
Construction and performance evaluation of the diagnostic nomogram. (**a**) The nomogram developed for predicting AMD risk based on the expression of *C3* and *HLA-DOA*. (**b**–**d**) Calibration curve, decision curve analysis (DCA), and receiver operating characteristic (ROC) curve evaluating the predictive accuracy of the model in the training set. (**e**–**h**) Corresponding model performance evaluation metrics in the independent validation set (GSE135092). * denotes statistical significance, indicating that the correlation/regression coefficient between HLA-DOA expression and Total points is statistically significant (*p* < 0.05).

Using the two biomarkers, we developed a nomogram to predict AMD onset. In this model, higher scores for *C3* and *HLA-DOA* elevated the total points, indicating a greater predicted risk of disease (Figure 3a). The calibration curve of the model closely followed the ideal 45 line, showing that its predictions matched the observed outcomes well. The Hosmer-Lemeshow test yielded a *p*-value of 0.983, indicating no significant deviation from a perfect fit. The model showed promising accuracy for AMD identification, supported by a high net benefit on DCA across most thresholds and an AUC of 0.795 on ROC analysis (Figure 3b–d). The nomogram maintained good predictive accuracy when applied to the validation dataset GSE135092 (Figure 3e), with an AUC of 0.702 (Figure 3f). The calibration curve showed close agreement between predicted and observed probabilities (Hosmer-Lemeshow test *p* = 0.553) (Figure 3g), and DCA confirmed positive net benefit across a range of threshold probabilities, supporting the generalizability of the model (Figure 3h). In conclusion, this line graph model demonstrates relatively excellent diagnostic and predictive capabilities, providing a reliable quantitative analysis tool for the molecular pathological classification of AMD and the study of the pathological mechanisms of retinal tissues. It also lays a theoretical foundation for further exploration of its expression characteristics in body fluids and clinical application transformation.

### 3.4. Investigation of the Potential Mechanisms of Action of Biomarkers

Enrichment analysis was performed on the biomarkers *C3* and *HLA-DOA*, which showed that biomarkers were negatively correlated with the enriched pathway oxidative phosphorylation, while positively correlated with multiple enriched pathways including complement and coagulation cascades, antigen processing and presentation, Toll-like receptor (TLR) signaling pathway, cytokine-cytokine receptor interaction, and NOD-like receptor signaling pathway (Figure S3 and Supplementary Tables S7 and S8). This correlation pattern suggests that the expression changes in *C3* and *HLA-DOA* are associated with energy metabolism disturbances in macular retinal cells in AMD. Furthermore, the dysregulation of AIR is correlated with these biomarkers, and alterations in complement activation, antigen presentation, and pro-inflammatory cytokine signaling pathways are also associated with *C3* and *HLA-DOA* expression levels, indicating that these genes may be involved in the formation of the macular inflammatory microenvironment and the progression of retinal damage.

**Figure 4:**
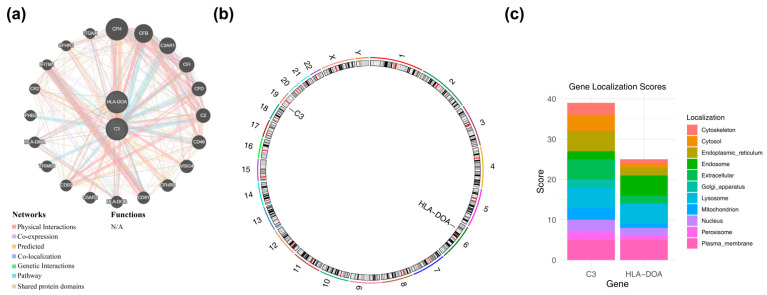
Functional interaction network and localization analysis of the identified biomarkers. (**a**) GeneMANIA functional association network for *C3* and *HLA-DOA*. (**b**) Chromosomal locations and (**c**) predicted subcellular localizations of the identified biomarkers.

Building on this, we performed further prediction to identify genes functionally linked to the biomarkers, which identified *CFH*, *CFB*, and *C3AR1*, among others, as functionally related candidates (Figure 4a). Chromosomal localization analysis revealed that *C3* and *HLA-DOA* were located on chromosomes 19 and 6, respectively (Figure 4b). Subcellular localization analysis indicated that *C3* might be localized to the endoplasmic reticulum, extracellular space, lysosome, and plasma membrane, while *HLA-DOA* might be localized to the endosome, lysosome, and plasma membrane (Figure 4c). Functional regulatory network analysis predicted interactions between the *C3* and *HLA-DOA* biomarkers and complement system components (*CFH*, *CFB*) as well as signaling molecules (*C3AR1*). Combined with the chromosomal and subcellular localization data, these bioinformatics predictions suggest a potential localized involvement of these genes in antigen presentation and complement-inflammatory cascades within the macular region. The definitive mechanistic contribution of these networks to retinal damage progression remains a subject for future experimental investigation.

### 3.5. Biomarkers and Immune Microenvironment Imbalance in AMD

**Figure 5:**
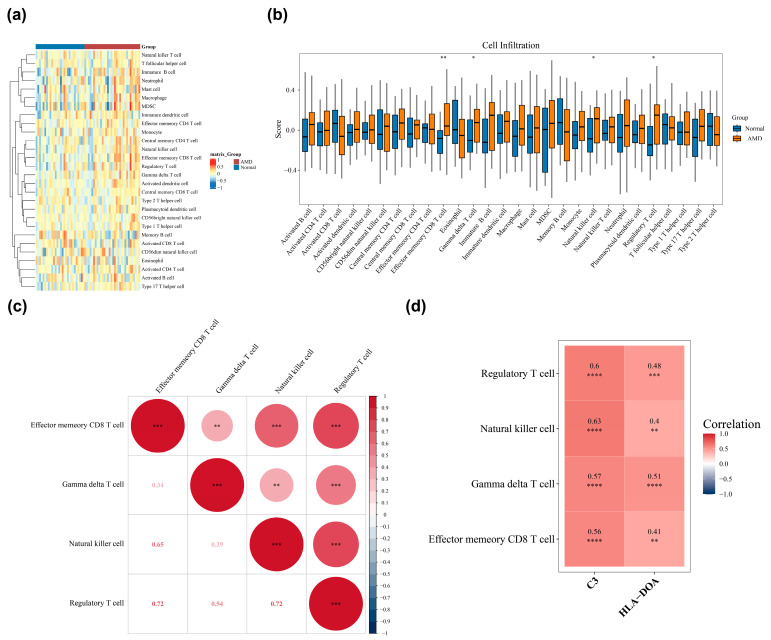
Immune microenvironment landscape and its correlation with biomarkers in AMD. (**a**) Heatmap illustrating the relative infiltration levels of significantly dysregulated immune cell populations. (**b**) Statistical comparison of the differentially infiltrated immune cell types between AMD and control groups. * *p* < 0.05, ** *p* < 0.01 indicate the levels of statistical significance for the differences in cell infiltration scores between AMD and control groups. (**c**,**d**) Correlation matrices depicting the relationships among dysregulated immune cells and their specific associations with *C3* and *HLA-DOA* expression. ** *p* < 0.01, *** *p* < 0.001, **** *p* < 0.00001 indicate the levels of statistical significance for the correlations between immune cells and between immune cells and *C3*/*HLA-DOA*.

Accordingly, an analysis of the retinal immune microenvironment was performed in AMD patients (Figure 5a). Significantly elevated enrichment scores were observed in the AMD group for several differentially infiltrated immune cells, such as effector memory CD8^+^ T (Tem) cells, gamma delta T cells, NK cells, and Tregs (vs. control, *p* < 0.05) (Figure 5b). Furthermore, the results of correlation analysis indicated that significant positive correlations existed among the aforementioned differential immune cell populations. Notably, Tregs exhibited the strongest positive correlations with both Tem and NK cells (both cor = 0.72, *p* < 0.001) among all immune cell pairs analyzed (Figure 5c). Significant positive correlations were identified between the biomarkers and the aforementioned immune cells through correlation analysis. *C3* demonstrated a particularly strong association with NK cells (cor = 0.63, *p* < 0.0001), and *HLA-DOA* was positively correlated with gamma delta T cells (cor = 0.51, *p* < 0.0001) (Figure 5d), suggesting that the expression levels of these two biomarkers are closely correlated with changes in the retinal immune microenvironment in AMD. Notably, their expression shows significant positive correlations with the infiltration levels of NK cells and gamma delta T cells, respectively, and aligns with trends observed in other immune cell populations. These statistically significant correlations highlight a quantitative association between biomarker expression and local immune infiltration. Such findings provide a data-driven basis for future mechanistic studies exploring the exact roles of these biomarkers in immune recruitment and inflammatory responses.

## 4. Discussion

Among people over 50 years old, AMD is the main cause of irreversible loss of central vision. Its pathogenesis involves complex immune dysregulation, including innate immune and adaptive immune responses. The complement system, a cornerstone of innate immunity, has been firmly established as a pivotal regulatory axis in the pathogenesis of AMD. As the central effector molecule of the complement cascade, *C3* represents one of the most unequivocally associated and thoroughly characterized genetic loci in AMD [31,32]. Extensive prior research has elucidated that the activation of *C3* (predominantly via the alternative pathway) and its functional cleavage fragments (*C3a*, *C3b*) are intimately associated with drusen deposition, RPE damage, and CNV in AMD patients [6,33]. Consequently, targeting the *C3*-mediated complement cascade has emerged as a highly promising therapeutic strategy for dry AMD [31]. While the role of innate immune mechanisms, exemplified by the complement system, in AMD pathogenesis is well-characterized, accumulating evidence indicates that adaptive immune cells can breach the blood-retinal barrier, infiltrate the otherwise immune-privileged retinal tissue, and drive disease progression via localized chronic inflammation [34]. However, the specific pathways through which adaptive immunity interacts with canonical innate immune molecules (e.g., *C3*) to regulate AMD progression, as well as the synergistic effects between adaptive immunity-related genes and *C3*, remain poorly defined.

In this context, we leveraged transcriptomic data integrated with machine learning to identify core biomarkers associated with the AIR in AMD. Furthermore, we investigated the crosstalk between established AMD-related innate immune molecules and adaptive immune regulatory factors in disease pathogenesis. Building on the well-established association between *C3* and AMD, this study further identifies *C3* and *HLA-DOA* as core biomarkers linked to adaptive immune dysregulation in AMD. A nomogram model constructed based on these two biomarkers demonstrated robust diagnostic efficacy for AMD, achieving an area under the curve (AUC) of 0.795 in the training set and 0.702 in the validation set. GSEA revealed that both biomarkers are positively correlated with pathways such as the complement and coagulation cascades, antigen processing and presentation, and TLR signaling, while being negatively correlated with the oxidative phosphorylation pathway. These pathways are all critical for mediating the crosstalk between innate and adaptive immunity. Notably, the expression levels of both *C3* and *HLA-DOA* showed significant positive correlations with multiple dysregulated immune cell subsets during AMD progression, suggesting their potential role as central hubs connecting innate immune activation with adaptive immune dysregulation in AMD pathogenesis. It is crucial to emphasize that the novelty of this work does not lie in discovering the *C3*-AMD association. Instead, the successful replication of this known association acts as a rigorous proof-of-principle for the bioinformatic pipeline. By effectively capturing an established pathogenic factor as a ‘positive control’, the employed modeling approach demonstrates high reliability and robustness in screening for novel immune targets. Building upon this validated methodological framework, the study further identifies *HLA-DOA* as a novel candidate, situating both biomarkers within the framework of adaptive immune dysregulation in AMD, and delineating the combined predictive value and synergistic regulatory mechanisms of *C3* and the adaptive immunity-related gene *HLA-DOA* in AMD immunopathology.

To elucidate the mechanistic basis underlying these observations, we examined the biological functions of each biomarker in detail. *C3*, a central effector of the complement system, is synthesized as a precursor protein that is proteolytically cleaved into α and β subunits. During complement activation, *C3* is further processed into bioactive fragments such as *C3a* and *C3b*. *C3*, significantly upregulated in AMD, correlated positively with immune pathways (complement/coagulation; antigen presentation) and negatively with oxidative phosphorylation. This is consistent with its dual functions: *C3a* initiates innate immunity, while *C3b* promotes opsonization/MAC assembly and bridges innate-adaptive immunity via antigen presentation [33]. Elevated levels of *C3* were also associated with increased infiltration of Tem cells, gamma delta T cells, NK cells, and Tregs, suggesting that *C3* may drive AMD progression by activating these immune pathways and cell populations, thereby promoting maladaptive immune responses. Upregulation of *C3* represents a critical step in activating the complement and coagulation cascades, thereby initiating key pathological processes in AMD [31]. The *C3b* fragment can covalently bind to the surface of RPE, leading to MAC assembly and subsequent RPE injury and dysfunction [35]. A chronic inflammatory microenvironment in the macula is driven, in part, by the anaphylatoxin *C3a*, which recruits and activates innate immune cells like monocytes and macrophages [32]. This inflammatory milieu subsequently primes the retina for dysregulation of adaptive immune responses. The positive correlation between *C3* and antigen processing and presentation pathways highlights a central mechanism by which *C3* may regulate adaptive immunity [36]. Specifically, *C3b* acts as a co-stimulatory signal that enhances dendritic cell processing and presentation of retinal autoantigens (e.g., lipofuscin), promoting the differentiation of naïve CD8^+^ T cells into Tem cells [37]. Consistent with this, we observed Tem cell enrichment in AMD samples. These activated Tem cells can directly induce RPE cytotoxicity via perforin and granzyme release and exacerbate local inflammation through IFN-γ secretion, establishing a self-sustaining cycle: *C3* upregulation → enhanced antigen presentation → Tem activation → retinal damage [38]. Furthermore, the positive correlation between *C3* and TLR signaling suggests synergistic amplification of inflammatory responses. Despite these pro-inflammatory cascades, compensatory anti-inflammatory mechanisms are also activated in AMD. Although Treg infiltration was increased in association with *C3* upregulation, under chronic *C3*-driven inflammation, Treg immunosuppressive function may be compromised [39], ultimately failing to restrain effector cell activity and exacerbating immune imbalance. Finally, the inverse correlation between *C3* and oxidative phosphorylation points to metabolic reprogramming as a key contributor. *C3* may promote a shift toward glycolysis in immune cells—a metabolic state that supports pro-inflammatory polarization [40,41]—thereby reinforcing immune-mediated damage at the metabolic level.

*HLA-DOA*, a key member of the MHC class II gene family, primarily functions to inhibit antigen presentation in B cells and contributes to immune homeostasis by modulating intercellular signaling [42]. In this study, we observed significant upregulation of *HLA-DOA*—which encodes HLA-DO, a negative regulator of MHC class II antigen presentation—in AMD tissues. Broad immune dysregulation, evidenced by associations with multiple immune pathways and cell infiltration, suggests its involvement in disrupting immune balance in AMD. This pattern was exemplified by *HLA-DOA*, which showed a distinct positive correlation specifically with antigen processing and presentation pathways. While this may appear counterintuitive given its canonical role in suppressing HLA-DM activity and attenuating antigen presentation [42], elevated *HLA-DOA* expression may not enhance presentation efficiency per se, but rather disrupt the kinetics of antigen presentation. Such dysregulation could lead to prolonged presentation of self-antigens or aberrant peptide selection, ultimately contributing to loss of immune tolerance, offering a plausible mechanism for the observed T-cell imbalance in AMD. Consistent with this hypothesis, *HLA-DOA* expression correlated positively with enriched Tem cells, which have been documented in the peripheral blood or local microenvironment of AMD patients [38]. These cells can directly damage RPE cells through secretion of cytokines such as IFN-γ. Furthermore, *HLA-DOA* upregulation correlated positively with complement/coagulation cascades and TLR signaling, indicating a link to innate immune hyperactivation. Given the established roles of these pathways in driving AMD pathogenesis, *HLA-DOA* may act synergistically with complement and TLR signaling to amplify inflammatory responses. *HLA-DOA* may act synergistically with these pathways to amplify inflammatory signaling. Additionally, *HLA-DOA* showed a positive correlation with gamma delta T cells, an innate-like T-cell subset noted for its IL-17 production. This finding further supports *HLA-DOA*’s involvement in non-conventional T-cell activation, thus potentially driving exacerbation of choroidal neovascularization and RPE injury [43]. Although Tregs were enriched in the AMD group, their immunosuppressive function may be compromised [5]. Upregulated *HLA-DOA* could further impair Treg functionality within the immune microenvironment, leading to inadequate suppression of effector T-cell responses and aggravated immune dysregulation. The positive association between *HLA-DOA* and cytokine-cytokine receptor interaction pathways underscores the formation of a pro-inflammatory network (e.g., IFN-γ, IL-17) that sustains a chronic inflammatory environment, contributing to RPE damage and CNV. Finally, the negative correlation between *HLA-DOA* and oxidative phosphorylation highlights a metabolic dimension: a shift toward glycolytic metabolism in immune cells may support their activation and sustained pro-inflammatory phenotype [40,41], thereby reinforcing the pathological process. While existing PPI networks and GeneMANIA functional integration models provide valuable indirect computational support for these interactions, these associations reflect systemic transcriptomic correlations. The specific downstream effector mechanisms require further functional validation.

To assess the functional equivalence of the human-specific biomarker *HLA-DOA* in murine models, this study quantified the mRNA expression level of its orthologous gene, *H2-Oa*. According to genomic database annotations, *H2-Oa* is the established ortholog of human *HLA-DOA* in mice. Functional annotation analysis revealed high conservation of molecular functions between *H2-Oa* and *HLA-DOA*. Both are predicted to possess “MHC class II protein complex binding activity” and “peptide antigen binding activity,” and are involved in identical biological processes, including “antigen processing and presentation of exogenous peptide antigen via MHC class II” and “regulation of T cell differentiation.” Regarding expression patterns, while detailed comparative data on their expression in retinal tissue are currently lacking, the NCBI Gene database indicates that *H2-Oa* is expressed in multiple mouse tissues, including the cornea. Consequently, despite potential subtle differences in the regulatory control of MHC class II-related molecules across species, the high degree of sequence homology and functional conservation supports the use of *H2-Oa* as a model for investigating the potential role of *HLA-DOA* as a biomarker in the immunopathology of AMD.

Our findings indicate that *C3* and *HLA-DOA* function through linked mechanisms, acting synergistically to bridge innate and adaptive immunity in AMD. Specifically, *C3*, as the core of the complement system, initiates the innate immune response, promotes the release of pro-inflammatory factors, and mediates early RPE damage [2]. Conversely, *HLA-DOA*, a key MHC class II molecule, regulates exogenous antigen presentation and contributes to the breakdown of immune tolerance. In essence, a synergistic paradigm emerges wherein *C3* drives the initiation of inflammation, and *HLA-DOA* orchestrates the subsequent adaptive immune imbalance; together, they jointly drive the continuous immunopathological progression of AMD. Furthermore, compared to recent high-impact transcriptomic studies on AMD—which have largely focused on singular pathological processes such as cellular senescence [44] or specific isolated immune subsets like follicular helper T cells [45]—this research offers a highly integrated perspective. To the best of current knowledge, this study is the first to systematically integrate the *C3* and *HLA-DOA* axis, adaptive immune responses, a predictive nomogram, and the Integrating the comprehensive immune microenvironment into a unified analytical framework. This multi-dimensional integration highlights the completeness of the current study, underscores the high predictive value of the constructed model, and provides a robust theoretical foundation with significant clinical translational potential for future multi-target personalized immunomodulatory therapies.

The selection of the laser-induced CNV mouse model for in vivo validation was driven by several critical scientific rationales. First, this model is currently the most classic and widely utilized in vivo system globally for simulating neovascular (wet) AMD. It effectively recapitulates core immunopathological processes, such as retinal inflammation and abnormal angiogenesis, making it highly suitable for the preliminary in vivo validation of key gene expression under inflammatory conditions [46]. Second, *C3* is highly conserved between mice and humans, demonstrating remarkable consistency in both expression and function [47]. Furthermore, the murine ortholog of *HLA-DOA*, *H2-Oa*, has been extensively validated in numerous studies to possess comparable functions in antigen presentation and immune regulation, serving as an ideal and widely accepted surrogate for evaluating the in vivo expression dynamics of this biomarker in mice [48,49]. Therefore, although this model cannot fully replicate the complex pathological features of human dry AMD and inherent species-specific differences exist, the primary objective of this animal experiment was not to perfectly duplicate the human disease. Rather, the fundamental motivation was to conduct a preliminary in vivo validation of the expression trends of these newly identified biomarkers in an active immune-inflammatory microenvironment. This approach provides an essential preliminary framework for subsequent, more in-depth mechanistic studies, fully justifying its scientific necessity, feasibility, and ethical rationale.

While this study provides initial evidence, via bioinformatics and animal experiments, for the potential of *C3* and *HLA-DOA* as immune-related biomarkers in AMD, several limitations should be noted. First, the mechanistic hypotheses are primarily derived from transcriptomic correlations and computational inference utilizing public databases. Although rigorous bioinformatic pipelines and functional network predictions were employed to provide indirect support, direct validation of the causal roles of *C3* and *HLA-DOA* in AMD pathogenesis remains a limitation. Functional experiments, such as in vitro gene knockout or overexpression studies in RPE cells or in vivo models, are currently lacking. Therefore, the findings presented herein should be strictly interpreted as significant correlations and potential associations rather than definitive causal relationships. Future investigations must incorporate these functional experiments to comprehensively confirm the active roles of these biomarkers in driving AMD progression. Second, regarding the specific disease stages at which these biomarkers operate, the laser-induced CNV model utilized in this study primarily simulates the choroidal neovascularization characteristic of wet AMD. Consequently, the validated high expression of *C3* and *HLA-DOA* in this model directly reflects their potential driving roles in the inflammatory and neovascular phases of wet AMD. For dry AMD (e.g., geographic atrophy), conclusions must be drawn with greater caution. While elevated expression of these genes is also observed in the GSE29801 dataset, which contains dry AMD samples—suggesting a potential involvement in broader atrophic pathological processes—the absence of functional validation in direct dry AMD cellular or animal models leaves their precise causal relationship in dry AMD unclear. Therefore, future investigations employing specific dry AMD models (such as oxidative stress-induced or NaIO3-treated models) are warranted to further validate their roles. Furthermore, species-specific differences between *HLA-DOA* and its murine ortholog *H2-Oa* may affect the translational relevance of the findings. Third, the current findings are predominantly based on transcriptomic and bioinformatic analyses of clinical samples, thereby providing a theoretical framework for the role of adaptive immunity in dry AMD. However, due to the lack of functional validation in preclinical animal models or cellular models specific to dry AMD (such as oxidative stress-induced or NaIO_3_-treated models), the causal contributions of these immune pathways to the pathophysiological processes of the disease cannot be definitively established at this stage. Future investigations utilizing specific in vitro and in vivo dry AMD models are strictly required to further validate these theoretical results and elucidate the detailed molecular mechanisms. Fourth, the analyses were conducted utilizing public transcriptomic datasets that lack comprehensive clinical metadata, including key variables such as patient age, sex, comorbidities (e.g., hypertension, diabetes, and cardiovascular diseases), precise disease duration, and prior treatment or medication history. These clinical factors can significantly influence the systemic and local immune status, gene expression profiles, and overall disease progression in patients with AMD. Consequently, the absence of these data may introduce unavoidable confounding effects on the biomarker screening, pathway enrichment, and immune infiltration results presented herein. Complete and systematic clinical data are crucial for accurately deciphering molecular mechanisms and enhancing the reliability and clinical applicability of the identified biomarkers. Future prospective studies must incorporate large-scale clinical cohorts with detailed medical annotations to adequately adjust for these confounding factors (e.g., age, comorbidities, and treatment history), thereby enabling a more precise elucidation of the roles of adaptive immunity-related genes in AMD and improving the translational value of these findings. Finally, as the biomarkers were derived from invasive retinal tissue samples, the developed nomogram, though not immediately applicable for non-invasive clinical screening, provides a theoretical framework for understanding AMD molecular mechanisms.

Future research should prioritize validating whether these transcriptomic signatures are detectable in more readily accessible clinical samples, such as aqueous humor or peripheral blood, to bridge the gap between tissue-based discoveries and non-invasive diagnostics. Concurrently, future studies should prioritize utilizing datasets with comprehensive clinical metadata or systematically controlling for these confounders in experimental design to more precisely delineate the role of immune-related genes in AMD pathogenesis. Building on this foundation, employing gene-editing technologies to further validate the central role of *C3* and *HLA-DOA* in immunoregulation, and integrating multi-omics data to deeply characterize their involved molecular networks, will establish a theoretical basis for developing personalized immunomodulatory therapies for AMD.

## 5. Conclusions

In summary, this study successfully establishes a robust transcriptomic and bioinformatic framework to explore adaptive immune response-related mechanisms in AMD. Through multidimensional machine learning approaches, *C3* and *HLA-DOA* were highlighted as key biomarkers significantly correlated with immune cell infiltration and inflammatory pathways within the macular microenvironment. The successful re-identification of the established biomarker *C3* serves as a solid proof-of-principle for the analytical pipeline, while simultaneously revealing the novel potential of *HLA-DOA*. Furthermore, the constructed nomogram demonstrates reliable predictive efficacy for assessing AMD risk. Although these findings provide valuable theoretical insights into the immunopathology of AMD and offer data-driven perspectives for immune-targeted diagnostic strategies, the definitive causal roles of these biomarkers remain to be fully established. Future research must prioritize functional experiments utilizing specific in vitro and in vivo models of dry AMD to elucidate the exact molecular mechanisms underlying these transcriptomic associations.

## Figures and Tables

**Table 1:** Primer sequences for qPCR.

Species	Gene Name	NCBI Accession No.	Forward Primer (5′→3′)	Reverse Primer (5′→3′)	Product Length (bp)
Mus	*C3*	NM_009778	CTTAGCGACCAAGTGCCAGA	CCGCAATGACTGTTGGTGTC	175
Mus	*H2-Oa*	NM_008206	ACACACGAATTTGACGGGGA	GGGCGAAGTCTCCAAACTCA	91
Mus	*Actb*	NM_007393	GGCTGTATTCCCCTCCATCG	CCAGTTGGTAACAATGCCATGT	150

## Data Availability

The datasets GSE29801 and GSE135092 supporting this study are available from the Gene Expression Omnibus (GEO) repository at https://www.ncbi.nlm.nih.gov/geo/, accessed on 8 October 2025.

## Supplementary Materials

The following supporting information can be downloaded at: https://www.mdpi.com/article/10.3390/biomedicines14051123/s1, Table S1: Subtype distribution of AMD samples in the GSE29801 dataset; Table S2: Adaptive immune response-related genes; Table S3: Differentially expressed genes (DEGs); Table S4: Candidate genes; Table S5: GO-enriched functions of candidate genes; Table S6: KEGG-enriched pathways of candidate genes; Table S7: Pathways enriched by *C3*; Table S8: Pathways enriched by *HLA-DOA*; Figure S1: (a) GO enrichment analysis. (b) KEGG enrichment analysis. The size of the circles represents the number of genes, and the color of the circles indicates the significance of pathway enrichment; Figure S2: (a) Penalty coefficient plot and cross-validation plot for parameter selection in LASSO analysis. As the lambda value increases, the number of independent variables decreases. The dashed line indicates the position of the optimal lambda value for LASSO. (b) Curves of classification results for accuracy and error rate in SVM-RFE analysis. These curves are used to evaluate changes in model performance with different numbers of features. (c) Boxplot of Boruta feature selection analysis results. The x-axis represents feature names, the y-axis denotes feature importance values, and different colors indicate distinct categories of features; Figure S3: GSEA of biomarkers. The top of the curve indicates the positions of different gene sets; the peak of the curve represents the enrichment level of the gene set. The red-blue gradient area denotes positive and negative enrichment directions: the closer the curve is to the top or bottom, the higher the degree of positive or negative correlation between the gene set and biomarker expression.

## Abbreviation

The following abbreviations are used in this manuscript

AMD	Age-related macular degeneration
RPE	retinal pigment epithelium
CNV	choroidal neovascularization
AIR	Adaptive immune response
GEO	Gene Expression Omnibus
AIR-RGs	adaptive immune response-related genes
DEGs	differentially expressed genes
MSigDB	Molecular Signatures Database
PPI	Protein–protein interaction
STRING	search tool for the retrieval of interacting genes/proteins
GO	Gene Ontology
KEGG	Kyoto Encyclopedia of Genes and Genomes
LASSO	least absolute shrinkage and selection operator
SVM-RFE	support vector machine-recursive feature elimination
DCA	Decision Curve Analysis
ROC	Receiver Operating Characteristic
AUC	Area Under the Curve
GSEA	Gene set enrichment analysis
GeneMANIA	Gene Multiple Association Network Integration Algorithm
qPCR	quantitative polymerase chain reaction
NIH	National Institutes of Health
RT-qPCR	Reverse transcription-quantitative polymerase chain reaction
TLR	Toll-like receptor
