# Supplementary figures and images for: Exploring Biomarkers and Mechanisms of Action of Adaptive Immune Response in Age-Related Macular Degeneration Based on Transcriptomics
Source: Biomedicines. 2026 May 15;14(5):1123. doi: 10.3390/biomedicines14051123 (PMC13204548; doi:10.3390/biomedicines14051123)

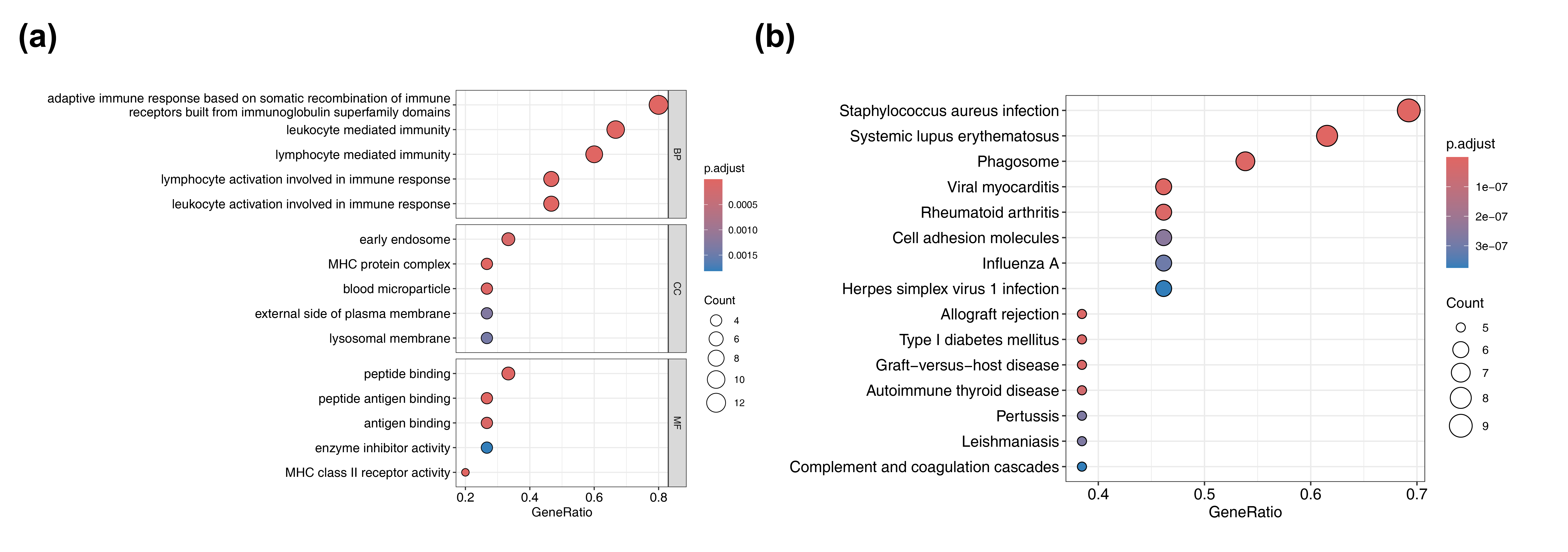

Supplement: Supplementary file 1 [file biomedicines-14-01123-s001.zip › Figure S1.tif]

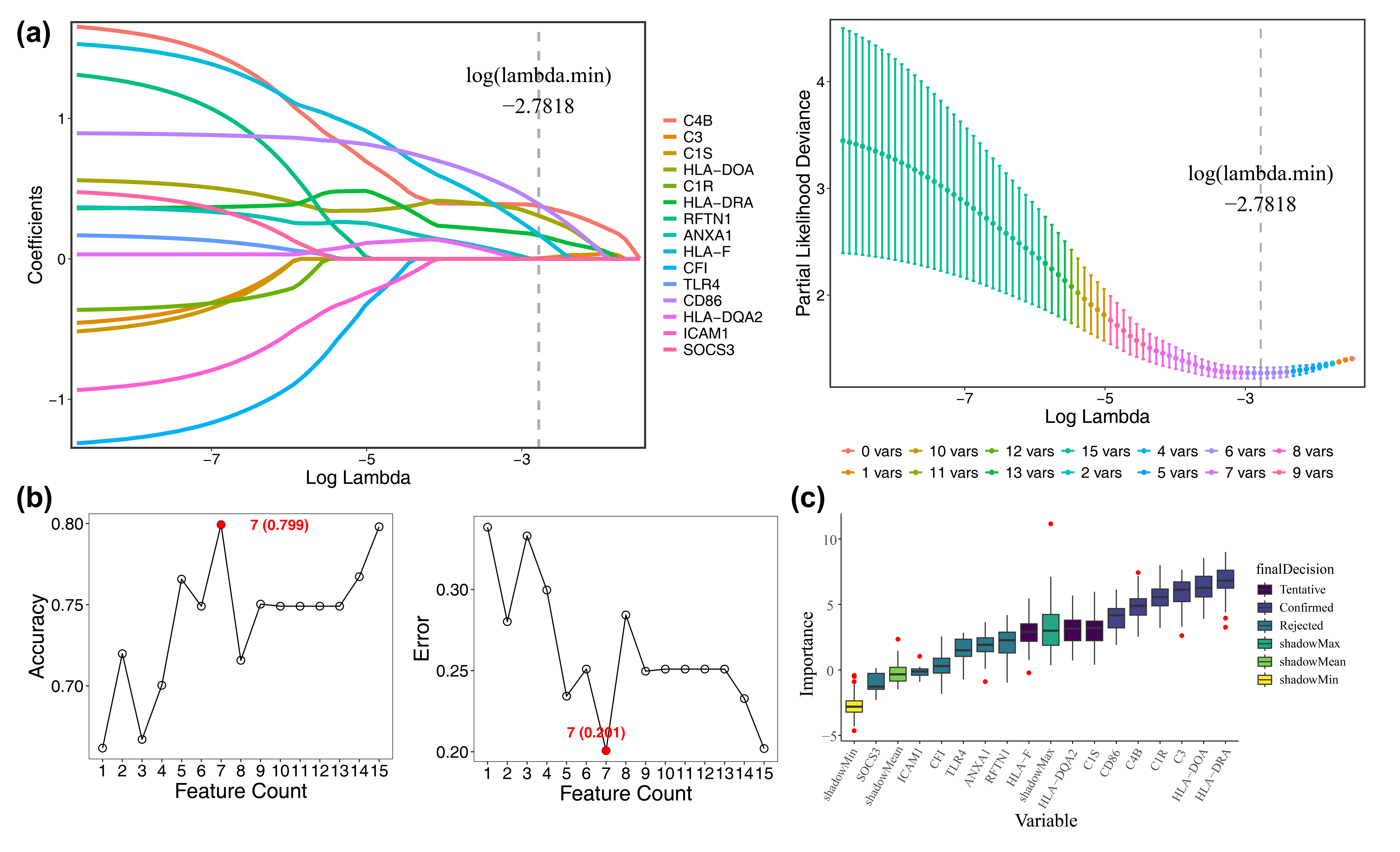

Supplement: Supplementary file 1 [file biomedicines-14-01123-s001.zip › Figure S2.tif]
